# The novel use of a nasal bridle system to remove a foreign body in the ear

**DOI:** 10.1002/ccr3.2269

**Published:** 2019-06-17

**Authors:** Robert Adrian Scott, Colin Wood, Imran Khan

**Affiliations:** ^1^ Department of Otolaryngology Queen Elizabeth University Hospital Glasgow UK

**Keywords:** bridle, ear, foreign body, magnet

## Abstract

Foreign bodies in the ear are a common presentation that prompt referral to Otolaryngology. We describe a technique that is safe and simple to perform for the removal of metallic foreign bodies in the ear. Case report of an elderly gentleman presenting with otalgia and a hearing aid battery lodged within bony external ear canal.

## 1 INTRODUCTION

Foreign bodies in the external auditory canal (EAC) are a common presentation to the Emergency Department (ED), often seen in children.^1^, ^2^ The type of foreign body and the difficulty of removal determines the rate of complications and indication for specialist referral.^1^, ^2^, ^3^, ^4^ Typical foreign bodies include cotton buds, parts of toys, earplugs, insects, and batteries. The EAC is an oval tube from the outer ear to the middle ear, composed of a cartilaginous outer third and bony inner two‐thirds. Due to its sigmoid shape, objects can readily become lodged, particularly at the bony isthmus. Techniques for retrieval in the ED are varied and include the use of grasping instruments, a Katz extractor, curved hooks, and irrigation.^3^ A battery foreign body requires urgent removal due to the risk of severe tissue damage of the EAC, tympanic membrane, middle ear structures, and hearing loss.^5^, ^6^

## 2 DISCUSSION

### 2.1 Case scenario

A 78‐year‐old gentleman presented with right otalgia. His family noticed he was holding the right side of his head and suspected his hearing aid was broken. He was noted to have multiple co‐morbidities including Alzheimer's disease, bilateral sensorineural hearing loss, atrial fibrillation, implantable cardiac pacemaker, and hypertension. Examination of the ear was painful and revealed a round battery (zinc‐air), lying obliquely within the EAC and lodged firmly at the bony isthmus.

### 2.2 Problem

Expedient removal of the battery is indicated due to the risk of liquefaction tissue necrosis from direct electrical current effects via alkaline caustic injury of tissues, prolonged local pressure, or from the leakage of the battery contents.^7^ Gaining compliance to facilitate the removal of the battery was extremely difficult due to the patient's advanced dementia and inability to cope when typical behavioral management strategies were employed.

### 2.3 Technique

**Figure 1:**
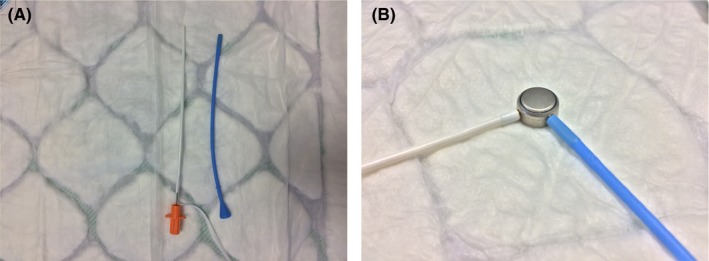
A, AMT Nasal Tube Retaining System. B, Magnetic rods enable atraumatic removal of battery

To successfully and swiftly retrieve the battery, we utilized a magnetic instrument that is readily available in our ED. We stock nasal bridle kits (Applied Medical Technology Nasal Tube Retaining System) that make use of two magnetic rods to secure nasogastric tubes to the nasal septum and prevent them being dislodged.^8^ Since a button battery is magnetic, the rods can swiftly and simply remove these foreign bodies. These conveniently shaped magnets were inserted into the EAC and engaged with the battery under direct vision. The impacted battery could be maneuvered, with enough strength, to safely dislodge and retrieve it out of the patient's ear (Figure 1A,B). No complications were observed. There was no visible injury to the external ear. The patient and their next of kin were very satisfied with the technique which maintained their comfort and compliance resulting in a successful outcome.

### 2.4 Considerations

This nasal bridle system is readily available and provides a simple and reliable method of removing metallic foreign bodies. To our knowledge, this technique has not been described and has now been applied to several patients referred to our department. Whilst we are aware of magnetic surgical instruments that have been designed for such an instance, their availability and cost are deterrent.^2^ This technique is sensitive to the comfort of the patient and can be successful even when there is inability to comply with other instrument removal or microscopic examination. Moreover, it avoids further trauma to the EAC, tympanic membrane, and middle ear structures. Sequelae in the longer term to observe for include ear canal stenosis in case of circumferential injury. The necessity of local anesthetic, sedation, or even general anesthesia in such a patient is often required if failure is encountered, and best circumvented given his co‐morbidities. This can similarly be applied to children with metallic foreign bodies in the ears or nasal cavity, particularly when traditional instruments are ineffective. We know of no contraindications to its use.

## 3 CONCLUSIONS

We describe a novel technique of foreign body removal from the ear, which is safe and simple to perform in the ED. This method utilizes equipment that is readily available and comparatively cost‐effective. With the potential to avoid an urgent general anesthetic procedure or specialist referral, we feel this technique is an invaluable addition to our armamentarium and encourage its use.

## CONFLICT OF INTEREST

None declared.

## AUTHOR CONTRIBUTION

RAS and CW: were involved with patient management. RAS: prepared the manuscript. RAS, CW, and IK: guided, edited, and proofread the final manuscript.
